# Hypothyroidism as a Predictor of Surgical Outcomes in the Elderly

**DOI:** 10.3389/fendo.2019.00258

**Published:** 2019-04-24

**Authors:** Marco Vacante, Antonio Biondi, Francesco Basile, Roberto Ciuni, Salvatore Luca, Salomone Di Saverio, Carola Buscemi, Enzo Saretto Dante Vicari, Antonio Maria Borzì

**Affiliations:** ^1^Department of General Surgery and Medical-Surgical Specialties, University of Catania, Catania, Italy; ^2^Cambridge Colorectal Unit, Cambridge University Hospitals NHS Foundation Trust, Cambridge, United Kingdom; ^3^Department of Clinical and Experimental Medicine, Specialization School in Geriatrics, University of Catania, Catania, Italy

**Keywords:** hypothyroidism, elderly, surgery, thyrotoxicosis, low T3 syndrome

## Abstract

There is a high prevalence of hypothyroidism in the elderly population, mainly among women. The most important cause is autoimmune thyroiditis, but also iodine deficiency, radioiodine ablation, and surgery may be responsible for hypothyroidism in elderly hospitalized patients. Thyroid-related symptoms are sometimes comparable to physiological manifestations of the aging process, and hypothyroidism may be related with many symptoms which can be present in critical patients, such as cognitive impairment, cardiovascular, gastrointestinal, and hematological alterations, and eventually myxedema coma which is a severe and life-threatening condition in older adults. Adequate thyroid hormone levels are required to achieve optimal outcomes from any kind of surgical intervention. However, only few randomized clinical trials investigated the association between non-thyroidal illness (or low-T3 syndrome), and adverse surgical outcomes, so far. The goal of this review is to discuss the role of thyroid function as a predictor of surgical outcomes in the elderly.

## Key Concepts

- The achievement of euthyroidism represents the goal before elective surgery, in order to prevent the risk of complications. In non-elective surgery, a careful risk-benefit evaluation in hypothyroid patients before surgical treatment is needed.
- The range of thyroid hormone levels in older patients may be different compared to that in younger subjects. Features of physiological aging may be occasionally confused with hypothyroidism in elderly patients.
- An adequate titration of LT4 in older patients is mandatory to attain appropriate serum TSH concentrations and avoid the risk of iatrogenic thyrotoxicosis.

## Introduction

Primary hypothyroidism is the most frequent pathological hormone insufficiency; its prevalence is approximately 10 times higher in women compared to men, and its incidence raises with age (1) (Table 1). The UK Whickham cohort study showed a mean annual incidence of hypothyroidism of 35 cases per 10,000 surviving women and 6 cases per 10,000 surviving men, during a follow-up of 20 years (2). The overall prevalence of hypothyroidism in the Third National Health and Nutrition Examination Survey (NHANES III) cohort was 4.6% (3). In iodine-sufficient countries, the prevalence of hypothyroidism ranges from 1 to 2%, rising to 7% in subjects aged between 85 and 89 years (4). A 5-year study carried out in Australia highlighted a prevalence of subclinical hypothyroidism of 5.0% (5). Chronic lymphocytic thyroiditis (or Hashimoto's thyroiditis) represents the most common cause of primary hypothyroidism, accounting for around 50% of all cases. Other causes are iodine deficiency, radioiodine ablation, and surgery, that may be responsible for hypothyroidism in elderly hospitalized patients (6). Administration of amiodarone, antibacterial solutions or lithium can also be responsible for thyroid insufficiency (7).

**Table 1:** Major modifications in the aging thyroid.

**Structural modifications**	
	↑Size microfollicles
	↑Colloid cysts
	↑Lymphocytes infiltration
	↑Number of nodules
	↑Fibrosis
**Hormonal modifications**	
	Normal FT4 levels (↓secretion ↓degradation)
	Low-limit range FT3 levels
	↑rT3 levels
	↑TSH levels (<6.0 μUI/ml, 97.5th percentile over 70 years; <7.5 μUI/ml, 97.5th percentile over 80 years)
	↓bioactive TSH / immunoreactive TSH

**Figure 1:**
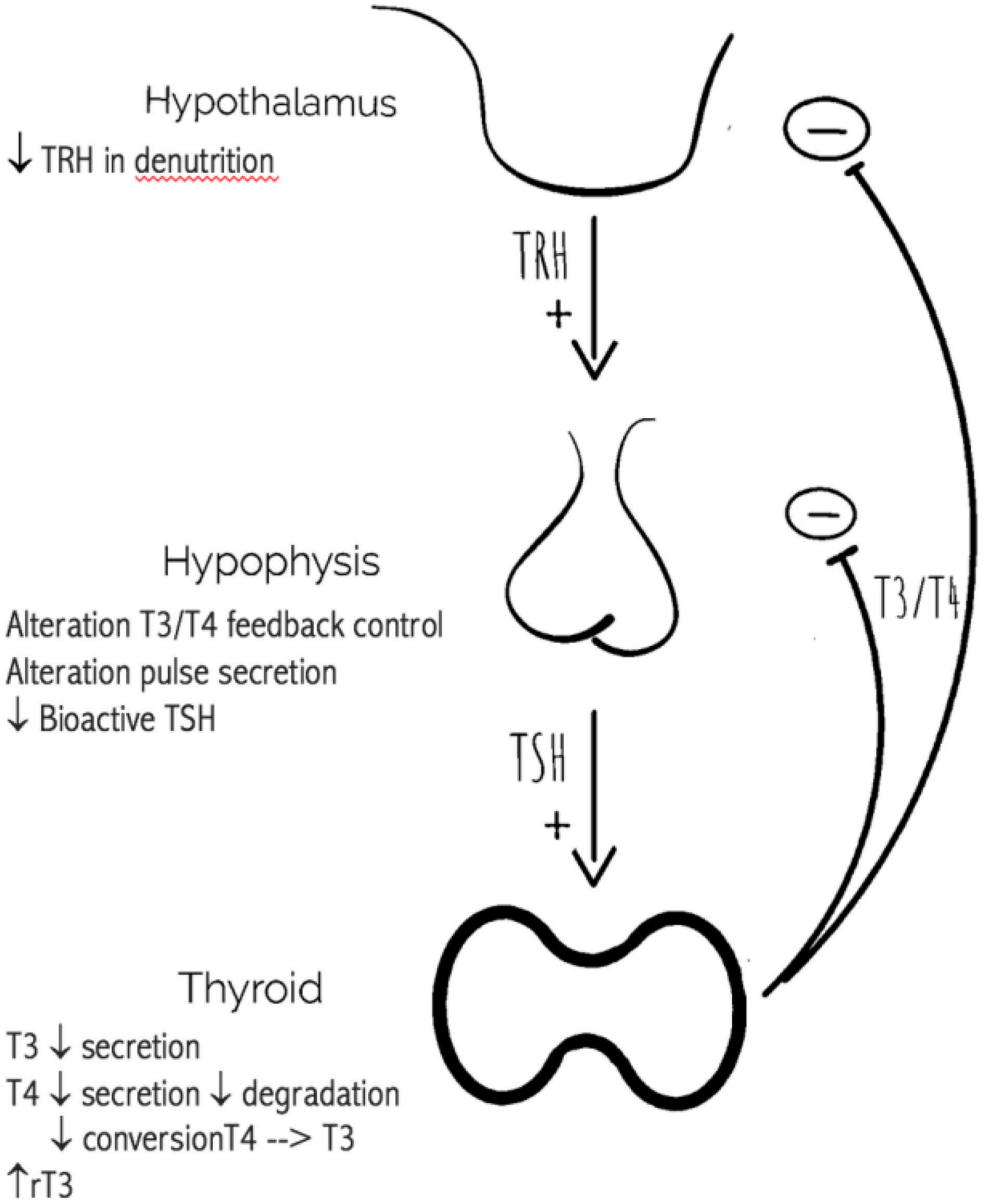
Age related changes in the hypothalamic-pituitary-thyroid axis.

Hypothyroidism may be classified as overt or subclinical (increased TSH with normal FT4 and FT3 levels). Subclinical hypothyroidism is common in elderly subjects and is associated with a number of clinical manifestations ranging from tiredness to cognitive impairment and coronary heart disease (8). Older patients require reduced dosages of levothyroxine to attain euthyroidism compared to younger patients, probably as a result of modifications in body composition or endocrine status occurring with age (9). The recent Institute for Evidence-Based Medicine in Old Age (IEMO) 80-plus thyroid trial aimed to investigate the effects of levothyroxine for 145 patients over 80 years with subclinical hypothyroidism (TSH ≥4.6 and ≤19.9 mU/L and FT4 within laboratory reference ranges). The results of this randomized clinical trial are expected to shed light on the multimodal effects of levothyroxine treatment in 80-plus subjects, highlighting benefits and potential adverse effects (10). The normal reference range of serum TSH in adult subjects is 0.4–4.5 mIU/L (11). In primary hypothyroidism it is possible to observe high TSH, low total T4, low FT4, high cholesterol (due to a reduction in the synthesis of LDL receptors), high creatine kinase (CK) levels due to skeletal muscle involvement and thyroid antibodies in case of Hashimoto's disease (12, 13). Secondary (or central) hypothyroidism (SH) is caused by a dysfunction of the pituitary gland or the hypothalamus, and is characterized by both decreased TSH secretion and low levels of thyroid hormones (Figure 1). SH can be classified into secondary and tertiary according to a pituitary or hypothalamic origin, respectively. Possible causes of SH include pituitary adenomas, and the subsequent surgical and/or radiotherapic treatment (14–16).

Non-thyroidal illness (NTI), or low-T3 syndrome, is a condition that occurs during acute stress or critical illness, due to a block in the peripheral conversion of thyroxine. NTI is a well-recognized negative prognostic factor in patients with severe acute disease. A recent study showed an association between preoperative hypothyroidism and post-operative arrhythmias in older patients, thus suggesting the utility of preoperative T3 evaluation and preoperative supplementation (17, 18). Low T3 syndrome is very common in the hospitalized older population, emerging as an independent predictor of short-term survival, thus suggesting FT3 determination as mandatory in the workup of these patients (19). The aim of this review was to summarize the role of thyroid function as a predictor of surgical outcomes in the elderly.

## Materials and Methods

To retrieve the articles, an extensive literature search was performed using the databases of Medline through PubMed, Scopus, and Google Scholar from January 2000 to September 2018. The search terms were “elderly,” “older adults,” “hypothyroidism,” “thyroid surgery.” Particular emphasis was given to implications of hypothyroidism on the surgical risk in elderly subjects. Manual search was also performed on numerous textbooks of medicine, endocrinology, and critical care.

### Clinical Features and Complications of Hypothyroidism in the Elderly

Thyroid-related symptoms are sometimes comparable to physiological manifestations of the aging process. In fact, signs, and symptoms of hypothyroidism are often less recognizable in elderly patients compared to younger subjects, thus posing diagnostic challenges (20). Nevertheless, hypothyroidism may be related with many symptoms which can be present in critical patients, such as cognitive impairment, cardiovascular, gastrointestinal, and hematological alterations, and eventually myxedema coma which is a severe and life-threatening condition in older adults. It is not possible to confirm a diagnosis of hypothyroidism based only on clinical symptoms, without TSH and FT4 assessment (21). In general, elderly subjects suffering from hypothyroidism may show classic symptoms, but complaints are often less specific than those described by younger hypothyroid patients (22). Doucet et al. compared the rate of 24 clinical symptoms of hypothyroidism between elderly patients and younger patients, and showed that fatigue and weakness were reported by more than 50% of the elderly patients, while increased sensitivity to cold, weight gain, paresthesiae, and muscle cramps were less common in the elderly (23). Carlè et al. compared the efficacy of hypothyroidism-associated symptoms in predicting overt hypothyroidism in different age groups, and observed that only dyspnea, fatigue and wheezing were more prevalent in elderly patients (24). Hearing loss, ataxia, and dysgeusia are neurological symptoms frequently described in hypothyroid older patients (25). Especially among elderly, neuropsychiatric symptoms such as memory loss or depression (26), dermatologic or rheumatologic disorders (27), are commonly described and it is difficult to related them to hypothyroidism. The list of signs in elderly with hypothyroidism may also comprise dry skin, hair loss, low heart rate, increased diastolic blood pression, pallor, and hoarseness (28). Cooper et al. observed that patients with subclinical hypothyroidism had a more elevated prevalence of symptoms as compared to controls with normal thyroid function (29). Another study by Kong et al. showed that the most common symptoms in women with subclinical hypothyroidism were fatigue (83%), weight gain (80%), and anxiety (50%) (30). Myxedema coma is a life-threatening condition due to hypothyroidism, which is characterized by a severe multiorgan failure (31). Myxedema coma is a rare disease, with an incidence of 0.22 per million per year in Europe (32). Most cases of myxedema coma occur in subjects 60 years and older (33) and are generally caused by precipitating factors that include exposure to cold, infections (i.e., pneumonia and urosepsis), withdrawal of thyroid supplements, and drugs (i.e., amiodarone or lithium) (34, 35). The diagnosis of myxedema coma is made on the combination of clinical manifestations and laboratory findings. The clinical presentation may include hypothermia, hypotension, bradycardia, congestive heart failure, hypoxaemia and hypercapnia, lethargy, and coma (36). Some patients show pericardial effusions, that are generally not hemodynamically significant. Laboratory assessment may show severe hypothyroidism, hypoglycemia, hyponatremia, and adrenal insufficiency (37). Myxedema coma represents an endocrine emergency with a mortality rate of nearly 40% (38). Major risk factors of mortality consist of older age, cardiovascular disease, and treatment with high-dose thyroid hormone (39).

### Preoperative Screening and Treatment Considerations

The effects of thyroid dysfunction are various and may complicate surgical procedures and post-operative recovery. Currently, there is no recommendation for routine screening to detect thyroidal disease in patients with no previous history of thyroid dysfunction. A preoperative TSH assessment should be performed in subjects with suspected thyroid disease or with known hypothyroidism (or hyperthyrodism) to optimize treatment before surgery (40).

There is general consensus about the utility to post-pone elective surgery until adequate treatment with thyroid hormone has achieved euthyroidism. At the preoperative stage, LT4 should be administered in a titrated manner to normalize the thyroid function. The optimal preparation period before elective surgery should range from 2 to 4 weeks. Patients older than 60 years, especially with coronary disease, should not be given full dose of LT4 at the beginning (40). In such patients, the starting dose is generally 25 μg per day, which increases every 2–6 weeks until the achievement of euthyroidism. In patients unable to take LT4 orally for more than 5 days after surgery, intravenous levothyroxine should be given at a dose between 60 and 80% of the oral dose (41).

### Implications of Hypothyroidism on the Surgical Risk

Preoperative recognition of hypothyroidism is crucial to reduce surgical and anesthesiological complications (41). Surgical trauma may influence the activity of the pituitary-thyroid axis, and thyroid hormones are secreted after surgery as a response to stress (42).

Anesthetic agents rather than surgical stress may be considered the main cause for the changes in plasma thyroid hormone concentrations during the intraoperative period (43). Many studies showed that adequate thyroid hormone levels are required to achieve optimal outcomes from any kind of surgical intervention (44). Correction of hypothyroidism, after replacement treatment, usually leads to the regression of pathophysiologic modifications due to low circulating thyroid hormone. Therefore, the achievement of euthyroidism represents the goal before elective surgery, in order to prevent the risk of complications. In non-elective surgery, a careful risk-benefit evaluation in hypothyroid patients before surgical treatment is needed. Only few randomized clinical trials investigated the association between NTI and adverse surgical outcomes so far (17) (Table 2).

**Table 2:** Main studies on the association between preoperative hypothyroidism and surgical outcomes.

**References**	**Patients**	**Drug administration**	**Main results**
Klemperer et al. (45)	142 patients undergoing CABG	Triiodothyronine *n* = 71 (Mean age 66 ± 10 years) or placebo *n* = 71 (Mean age 68 ± 9 years)	↑cardiac output ↓systemic vascular resistance No changes in post-operative mortality and morbidity
Worku et al. (18)	821 patients undergoing cardiac surgery Euthyroid *n* = 682 (Mean age 65.7 years) Hypothyroid *n* = 77 (Mean age 63.9 years)	None	Preoperative hypothyroidism was associated with post-operative atrial fibrillation
Cerillo et al. (46)	806 patients undergoing CABG Mean age 67.5 ± 9.6 years	None	Low T3 is a strong predictor of death and low cardiac output in CABG patients
Park et al. (47)	260 patients undergoing CABG Euthyroid *n* = 224 (Mean age 65.3 ± 9.4 years) SCH *n* = 36 (Mean age 65.4 ± 11.4 years)	None	↑post-operative atrial fibrillation
Jaimes et al. (48)	626 patients undergoing first-time isolated myocardial revascularization surgery Euthyroid *n* = 313 (Mean age 63 years) Hypothyroid *n* = 313 (Mean age 68 years)	None	Hypothyroidism is a risk factor for the onset of post-operative fibrillation

*CABG, coronary artery bypass grafting; SCH, subclinical hypothyroidism*.

A study by Park et al. did not show significant differences between patients with subclinical hypothyroidism and euthyroid patients undergoing a cardiovascular surgery procedure, as regards respiratory and cardiovascular complications, wound problems, leg infection, mediastinitis, and delirium. It was noteworthy that in the subclinical hypothyroidism group there was an increase in the rate of post-operative atrial fibrillation (47). Another study reported an association between preoperative hypothyroidism and post-operative atrial fibrillation in young-old patients, thus suggesting that preoperative hypothyroidism could be helpful for selecting those patients who would take advantage from preoperative replacement therapy in the prevention of post-operative atrial fibrillation (18). Furthermore, it has been observed a strong association between NTI at admission and increased risk of post-operative myocardial dysfunction and death in subjects undergoing coronary artery by-pass grafting (46).

A study by Weinberg et al. reported the effects of anesthesia and surgery in 59 hypothyroid patients compared with 50 euthyroid patients. The two groups did not show significant differences as regards duration of surgery or anesthesia, lowest temperature and blood pressure recorded during surgery, time to extubation, incidence of arrhythmias, need for vasopressors, fluid and electrolyte imbalances, sepsis, pulmonary and myocardial infarction, bleeding complications, or time to hospital discharge. After the analysis of subsets of thyroxine levels (thyroxine level <1.0 μg/dL, 1.0 to <3.0 μg/dL, and >3.0 μg/dL), the authors concluded that there was no evidence to post-pone surgery until the correction of mild or moderate hypothyroidism, whereas there was poor evidence to make a recommendation for patients with severe hypothyroidism (49).

Patients with hypothyroidism show slower drug metabolism and are exposed to the risk of an overdose of anesthetics and other medications used during the surgical treatment (50). The anesthesiological management of hypothyroid patients may face important clinical challenges, such as the presence of impaired baro-receptor reflex mechanism, depressed myocardial function, depressed ventilatory drive, and low glycaemia (51). There is no general consensus about surgery planning time for mild or moderate hypothyroidism as concerns anesthesia practice (52). However, in hypothyroid patients low-dose regional anesthesia could represent an option for minor surgery procedures (52). There is evidence that spinal, epidural or thiopental anesthesia could have low effects on thyroid hormones compared to general anesthesia; thus these methods should be taken into account in patients with thyroid function disorders, according to the type of surgical intervention needed.

## Conclusions

It is recommended to post-pone elective surgery in elderly patients with hypothyroidism until an euthyroid state is achieved. If patients need urgent or emergent surgery, it is recommended to proceed with surgery only if they have mild or moderate hypothyroidism. Replacement therapy should be started preoperatively and there should be growing attention to the possible occurrence of minor post-operative complications in hypothyroid patients. As suggested by the American Thyroid Association (ATA), the treatment in elderly patients should be initiated at low doses with slow titration based on serum TSH evaluation. Elderly patients show higher normal serum TSH ranges; thus, higher serum TSH targets may be necessary as a patient ages. The suggested target serum TSH in people age 70–80 years is 4–6 mIU/L (8). Further clinical trials assessing surgical management in older hypothyroid patients are firmly required.

## Author Contributions

MV, AMB, AB and FB conceived the review. MV and AMB wrote the manuscript and realized the figures and tables. SDS, SL, CB, RC and ESDV performed the literature search and critically revised the manuscript for important intellectual content.

### Conflict of Interest Statement

The authors declare that the research was conducted in the absence of any commercial or financial relationships that could be construed as a potential conflict of interest.
